# No agreement of mixed venous and central venous saturation in sepsis, independent of sepsis origin

**DOI:** 10.1186/cc9348

**Published:** 2010-11-29

**Authors:** Paul A van Beest, Jan van Ingen, E Christiaan Boerma, Nicole D Holman, Henk Groen, Matty Koopmans, Peter E Spronk, Michael A Kuiper

**Affiliations:** 1 Department of Anesthesiology, University Medical Center Groningen, Hanzeplein 1, Groningen, 9700 RB, The Netherlands; 2 Department of Intensive Care Medicine, Martini Hospital, Van Swietenplein 1, Groningen, 9700 RM, The Netherlands; 3 Department of Intensive Care Medicine, Medical Center Leeuwarden, Henri Dunantweg 2, Leeuwarden, 8901 BR, The Netherlands; 4 Department of Epidemiology, University Medical Center Groningen, Hanzeplein 1, Groningen, 9700 RB, The Netherlands; 5 Department of Intensive Care Medicine, Gelre Hospital Apeldoorn, Albert Schweitzerlaan 31, Apeldoorn, 7300 DS, The Netherlands; 6 Department of Intensive Care Medicine L.E.I.C.A, Academic Medical Center, Meibergdreef 9, Amsterdam, 1105 AZ, The Netherlands

## Abstract

**Introduction:**

Controversy remains regarding the relationship between central venous saturation (ScvO_2_) and mixed venous saturation (SvO_2_) and their use and interchangeability in patients with sepsis or septic shock. We tested the hypothesis that ScvO_2 _does not reliably predict SvO_2 _in sepsis. Additionally we looked at the influence of the source (splanchnic or non-splanchnic) of sepsis on this relationship.

**Methods:**

In this prospective observational two-center study we concurrently determined ScvO_2 _and SvO_2 _in a group of 53 patients with severe sepsis during the first 24 hours after admission to the intensive care units in 2 Dutch hospitals. We assessed correlation and agreement of ScvO_2 _and SvO_2_, including the difference, i.e. the gradient, between ScvO_2 _and SvO_2 _(ScvO_2 _- SvO_2_). Additionally, we compared the mean differences between ScvO_2 _and SvO_2 _of both splanchnic and non-splanchnic group.

**Results:**

A total of 265 paired blood samples were obtained. ScvO_2 _overestimated SvO_2 _by less than 5% with wide limits of agreement. For changes in ScvO_2 _and SvO_2 _results were similar. The distribution of the (ScvO_2 _- SvO_2_) (< 0 or ≥ 0) was similar in survivors and nonsurvivors. The mean (ScvO_2 _- SvO_2_) in the splanchnic group was similar to the mean (ScvO_2 _- SvO_2_) in the non-splanchnic group (0.8 ± 3.9% vs. 2.5 ± 6.2%; *P *= 0.30). O_2_ER (*P *= 0.23) and its predictive value for outcome (*P *= 0.20) were similar in both groups.

**Conclusions:**

ScvO_2 _does not reliably predict SvO_2 _in patients with severe sepsis. The trend of ScvO_2 _is not superior to the absolute value in this context. A positive difference (ScvO_2 _- SvO_2_) is not associated with improved outcome.

## Introduction

Global tissue hypoxia as a result of systemic inflammatory response or circulatory failure is an important indicator of serious illness preceding multiple organ failure. The development of organ failure predicts outcome of the septic patient [1]. Unrecognized and untreated global tissue hypoxia increases morbidity and mortality: decreased mixed venous saturation (SvO_2_) values predict poor prognosis in septic shock [2-4]. Controversy, however, remains: there is no clear evidence that guiding hemodynamic optimization by monitoring central venous saturation (ScvO_2_) or SvO_2 _is useful in all patients with sepsis or septic shock, especially in the intensive care unit (ICU). The controversy includes the interchangeability of ScvO_2 _and SvO_2 _[5,6]. Also, in patients with a splanchnic cause of sepsis, ScvO_2 _may be normal, whereas the SvO_2 _may be decreased because of elevated metabolic demand. On the other hand, owing to sepsis-related vasodilatation (also in the digestive tract) leading to diminished oxygen consumption, SvO_2 _may be normal [7]. This could mean that the 5% difference between ScvO_2 _and SvO_2 _is not as consistent in sepsis as postulated earlier [8,9]. Nevertheless, recently, an association between a positive O_2 _gradient (ScvO_2 _- SvO_2 _≥0) and ICU survival in critically ill patients was described [10]. Therapy aimed at increasing this gradient could mean improved survival. However, this demands measurement of both ScvO_2 _and SvO_2_.

We tested the hypothesis that ScvO_2 _does not reliably predict SvO_2 _in sepsis; that is, a consistent 5% difference between ScvO_2 _and SvO_2 _does not exist. We also looked at the possible relationship between a positive difference between ScvO_2 _and SvO_2 _(ScvO_2 _- SvO_2_) and ICU survival. In a secondary analysis, we tested the hypothesis whether the relationship between ScvO_2 _and SvO_2 _is independent of sepsis origin or not.

## Materials and methods

### Setting

We studied ICU populations in two teaching hospitals: the Martini Hospital (MH) (Groningen, The Netherlands), where the ICU is a 14-bed 'closed format' mixed medical/surgical ICU department, and the Medical Center Leeuwarden (MCL) (Leeuwarden, The Netherlands), where the ICU is a 16-bed 'closed format' mixed medical/surgical ICU, including cardiothoracic patients. The study was approved by both local ethics committees. Informed consent was obtained in all cases from the patient or the patient's legal representative.

### Patients and data collection

This prospective observational study included patients (at least 18 years old) with sepsis or septic shock according to international criteria [11] between January and September 2009. Only patients in whom there was a clinical indication for additional hemodynamic monitoring using a pulmonary artery catheter (PAC) (Criticath SP 5507 H TD; Becton Dickinson, Singapore) or a continuous cardiac output (CCO) catheter (Arrow Deutschland GmbH, Erding, Germany) were included. The catheter was inserted in an internal jugular vein or subclavian vein in accordance with standard procedure. Position was confirmed by the presence of pulmonary artery pressure tracings and chest radiography. No complications other than transient arrhythmias were observed during the insertion of any catheter. Primary data, including hemodynamic parameters, were collected at 6-hour intervals (T0, T1, T2, T3, T4) during the first 24 hours after acute ICU admission. Standard blood samples (2 mL) were drawn simultaneously from distal (pulmonary artery) and proximal/side (superior caval vein) ports from the PAC or CCO catheter. To avoid falsely high readings because of aspiration of pulmonary capillary blood, aspiration was done gently to avoid high negative pressure when blood samples were taken. We took blood from the proximal port of the catheter as representative of central venous blood [6,8,10]. We did not use any continuously measured values of the catheter itself in the cases in which a CCO catheter was used. Only patients with a complete series of five paired measurements were finally included. Also, arterial blood samples, including serum lactate, were obtained. All blood samples were analyzed by a co-oximeter (Radiometer ABL800 flex; Radiometer, Copenhagen, Denmark). The Acute Physiology and Chronic Health Evaluation II (APACHE II) score after 24 hours of ICU admission was collected [12].

### Statistical analysis

Analysis was conducted for the total population, and for secondary analysis, the population was divided into two groups: patients with a splanchnic source of sepsis and patients with a non-splanchnic source of sepsis. We calculated a sample size of 200 paired samples to detect an absolute difference between ScvO_2 _and SvO_2 _in a two-sided test with a 0.05 type I error and a 95% probability in case of standard deviation of 10% [13,14]. Statistical tests were two-tailed and performed by the statistical package for the social sciences (SPSS 16.0.1 for Windows; SPSS Inc., Chicago, IL, USA) or MedCalc software (version 11.2.1; MedCalc Software, Mariakerke, Belgium). The latter were used for comparing receiver operating characteristic (ROC) curves. GraphPad software (Prism 5.0; GraphPad Software, Inc., La Jolla, CA, USA) was used for graphics. Measurements were not independent but were clustered within each patient. All data were tested for normal distribution with the Kolmogorov-Smirnov test before further statistical analysis. Differences between the two groups were assessed by using the Student *t *test in case of normal distribution or the χ^2 ^test. For each time point T0 toT4, (ScvO_2 _- SvO_2_) was calculated including the average difference per patient. The agreement between absolute values of ScvO_2 _and SvO_2 _and the agreement of the changes of these values were assessed by the mean bias and 95% limits of agreement ([mean bias ± 1.96] × standard deviation) as described by Bland and Altman [15]. The χ^2 ^test was used to establish significance between the number of survivors and non-survivors. Spearman correlations for assessing possible factors affecting (ScvO_2 _- SvO_2_) were determined: at each time point, (ScvO_2 _- SvO_2_) was compared with hemodynamic and perfusion variables.

For secondary analysis, we also calculated the mean (ScvO_2 _- SvO_2_) per group, and these values were compared by using Student unpaired *t *test. Additionally, the influence on outcome of O_2_ER was determined because (ScvO_2 _- SvO_2_) did correlate with O_2_ER in the secondary analysis. SvO_2 _and arterial oxygen saturation (SaO_2_) were used in the calculation of the systemic oxygen extraction ratio (O_2_ER). ROC curves were used for the assessment of sensitivity and specificity of O_2_ER in predicting in-hospital mortality. Data were displayed as mean ± standard deviation. Statistical significance was assumed at a *P *value of less than 0.05.

## Results

We enrolled 56 patients, of whom 3 patients were excluded because of lack of data (technical problems). We evaluated data from 53 patients with sepsis. Altogether, 265 paired blood samples were obtained. Baseline characteristics and outcome of the total population and both groups are shown in Table 1. Length of stay in the ICU (LOS_ICU_) was 12 ± 10 days, and length of stay in the hospital (LOS_HOSP_) was 25 ± 18 days.

**Table 1:** Baseline characteristics and outcome

Variable	Total population (*n *= 53)	Splanchnic group (*n *= 25)	Non-splanchnic group (*n *= 28)	*P *value^a^
Age, years	66 ± 12	66 ± 12	66 ± 13	0.46
Central venous pressure, mm Hg	12 ± 6	11 ± 5	14 ± 6	0.06
Mean arterial pressure, mm Hg	66 ± 10	65 ± 12	66 ± 9	0.65
ScvO_2_, percentage	72.0 ± 10.0	73.7 ± 10.5	70.6 ± 9.6	0.29
SvO_2_, percentage	71.8 ± 10.6	75.2 ± 9.9	68.6 ± 10.5	0.03^b^
Lactate, mmol/L	3.5 ± 3.5	3.8 ± 3.8	3.5 ± 3.2	0.33
Arterial pH	7.30 ± 0.10	7.29 ± 0.10	7.29 ± 0.12	0.43
Hematocrit, percentage	30.1 ± 5.7	30.2 ± 6.1	32.1 ± 5.7	0.59
APACHE II score	26.6 ± 7.6	25.3 ± 7.3	28.7 ± 7.8	0.24

Hospital mortality, percentage	26.5	29.2	24.0	0.56

Data are presented as mean ± standard deviation unless otherwise indicated. ^a^Splanchnic group versus non-splanchnic group. ^b^Statistically significant difference. APACHE II, Acute Physiology and Chronic Health Evaluation II; ScvO_2_, central venous oxygen saturation; SvO_2_, mixed venous oxygen saturation.

**Figure 1:**
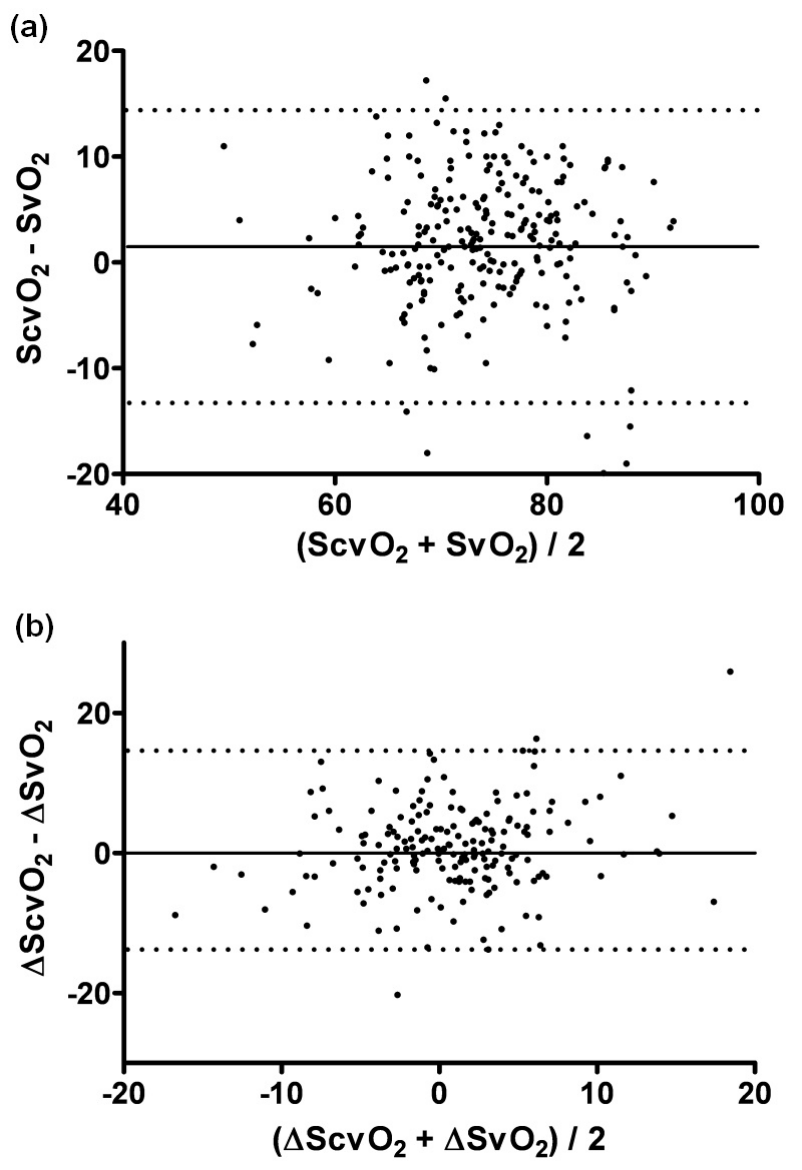
**Bland and Altman plot showing the agreement between (a) ScvO_2 _and SvO_2 _(bias 1.7, 95% limits of agreement from -12.1 to 15.5) and in (b) changes in ScvO_2 _and SvO_2 _(bias 0.6, 95% limits of agreement from -13.4 to 14.6)**. ScvO_2_, central venous saturation; SvO_2_, mixed venous saturation.

**Figure 2:**
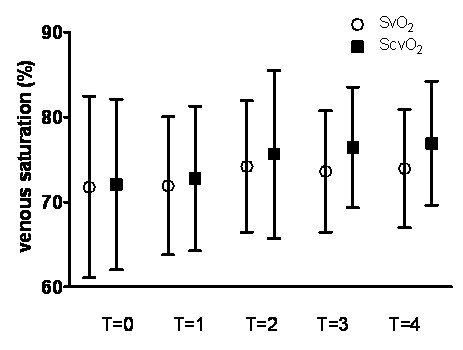
**Mean mixed venous saturation (SvO_2_) and central venous saturation (ScvO_2_) values at different time points**. ScvO_2 _is consistently higher than SvO_2 _without statistical difference (paired *t *test; all *P *> 0.05).

The ScvO_2 _overestimated the SvO_2 _by a mean bias (or absolute difference) of 1.7% ± 7.1% in the total population. The 95% limits of agreement were wide (-12.1% to 15.5%; Figure 1a). Figure 2 illustrates this: mean ScvO_2 _and mean SvO_2 _values are shown at each time point. Results at time point T = 0 and at different time points were similar, including wide limits of agreement (data and plots not shown). Bias between changes of ScvO_2 _and SvO_2 _was 0.6% ± 7.1% in the total population, with 95% limits of agreement of -13.4% to 14.6% (Figure 1b). Results were similar at time point T = 0 and at different time points, including wide limits of agreement (data and plots not shown).

### Differences between survivors and non-survivors

**Figure 3:**
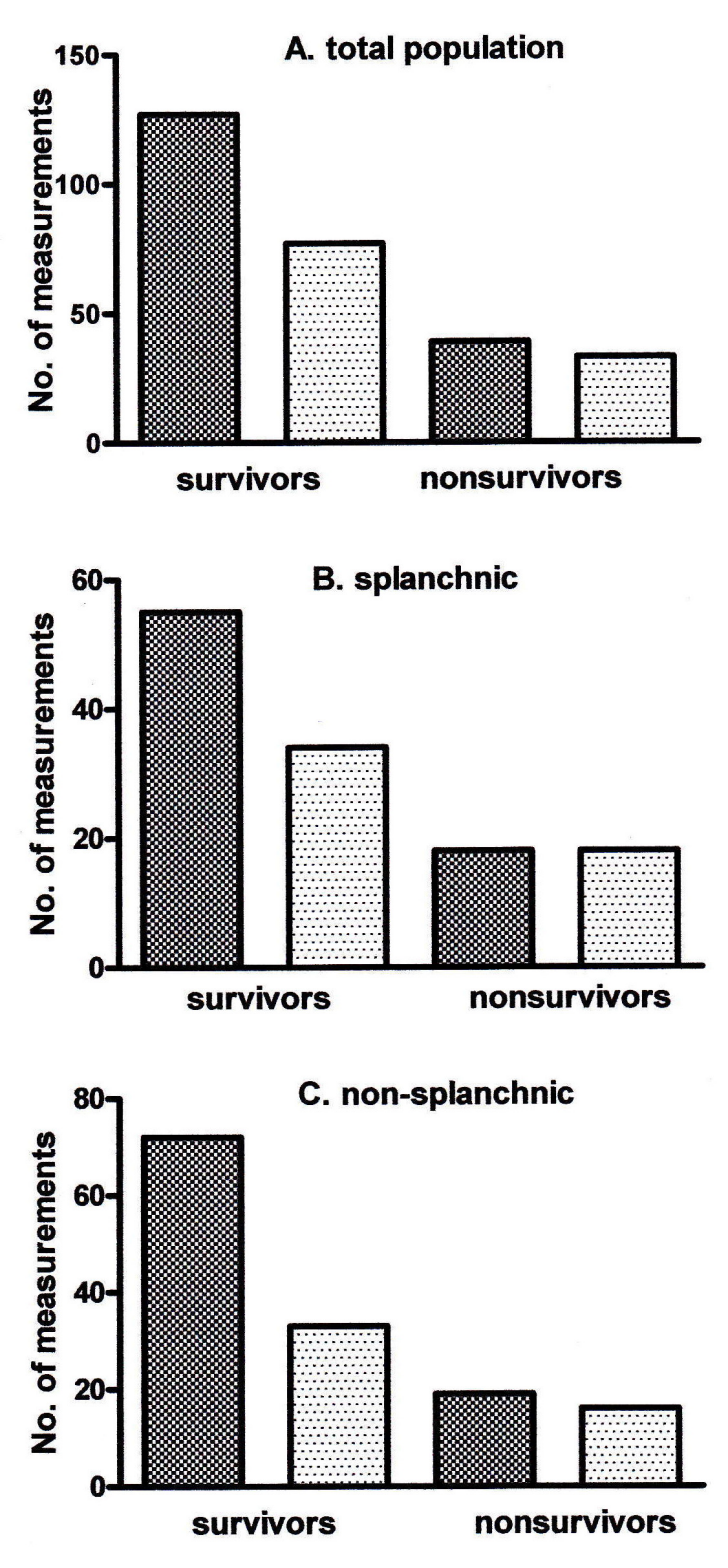
**Number of paired measurements resulting either in an (ScvO_2 _- SvO_2_) of at least 0 (dark bars) or in an (ScvO_2 _- SvO_2_) of less than 0 (light bars)**. There was no significantly different distribution of (ScvO_2 _- SvO_2_) between survivors and non-survivors in **(a) **the total population (*P *= 0.13), **(b) **the splanchnic group (*P *= 0.23), or **(c) **the non-splanchnic group (*P *= 0.13). The χ^2 ^test was used to establish significance between the number of survivors and non-survivors. ScvO_2_, central venous saturation; SvO_2_, mixed venous saturation.

As ScvO_2 _of 70% has been used as a target for guided therapy in septic patients [4], we evaluated the frequencies of ScvO_2 _values below 70% in both survivors and non-survivors. Of all ScvO_2 _measurements in survivors, 15% fell below 70%, whereas in non-survivors, 47% of all ScvO_2 _measurements fell below 70% (*P *< 0.01). Assuming a 5% difference between ScvO_2 _and SvO_2 _[1], we also evaluated the frequencies of SvO_2 _values below 65% in both survivors and non-survivors. Of all measurements in survivors, 7% fell below 65%, whereas in non-survivors, 27% of all SvO_2 _measurements fell below 65% (*P *< 0.01). Figure 3 shows the number of paired measurements resulting in either an (ScvO_2 _- SvO_2_) of at least 0 or an (ScvO_2 _- SvO_2_) of less than 0. There was no significant different distribution of (ScvO_2 _- SvO_2_) between survivors and non-survivors (*P *= 0.13).

### Influence on difference between ScvO_2 _and SvO_2 _(ScvO_2 _- SvO_2_)

The difference between ScvO_2 _and SvO_2 _was dependent on the level of ScvO_2 _when values of less than 60%, 60% to 70%, 70% to 80%, and greater than 80% were analyzed separately. The mean (ScvO_2 _- SvO_2_) values were 8.9%, 1.0%, 2.4%, and 4.2%. Owing to a low incidence (4.9%) of low ScvO_2 _values (< 60%), we did not assess statistics on these differences. Assessment of Spearman correlation coefficients did not show any relation between cardiac output, cardiac index, dopamine (μg/kg per minute), norepinephrine (μg/kg per minute), mean arterial blood pressure, arterial saturation, hemoglobin, hematocrit, pH, or lactate levels and (ScvO_2 _- SvO_2_) (all *P *> 0.05). O_2_ER correlated significantly with (ScvO_2 _- SvO_2_) at all time points (all *P *< 0.01).

### Differences between groups

Secondary analysis showed that 25 patients presented with a splanchnic source of sepsis and 28 patients presented with a non-splanchnic source of sepsis. Thirty patients (15 splanchnic and 15 non-splanchnic) were enrolled in the MCL, and 23 (10 splanchnic and 13 non-splanchnic) patients were enrolled in the MH. The sources of sepsis in the non-splanchnic group were mainly pneumonia (*n *= 16; 57%) and infection of the urogenital tract (*n *= 5; 18%). Other sources were meningitis, arthritis, epiglottitis, endocarditis, and infected soft tissue. At baseline, SvO_2 _(75.2% ± 9.9% versus 68.6% ± 10.5%; *P *= 0.03) was different between groups. There was no significant difference between the mean (ScvO_2 _- SvO_2_) of the two groups: splanchnic, 0.8% ± 3.9% versus non-splanchnic, 2.5% ± 6.2% (*P *= 0.30). Biases between ScvO_2 _and SvO_2 _were 0.7% ± 6.3% (95% limits of agreement of -11.7% to 13.1%) in the splanchnic group and 2.6% ± 7.5% (95% limits of agreement of -12.2% to 17.4%) in the non-splanchnic group. Biases between changes in ScvO_2 _and SvO_2 _were 0.9% ± 7.9% (95% limits of agreement of -14.5% to 16.3%) in the splanchnic group and 0.3% ± 6.5% (95% limits of agreement of -12.4% to 13.0%) in the non-splanchnic group (plots not shown). The difference between ScvO_2 _and SvO_2 _was dependent on the level of ScvO_2 _when values of less than 60%, 60% to 70%, 70% to 80%, and greater than 80% were analyzed separately. The mean (ScvO_2 _- SvO_2_) values were 12.3%, 2.1%, 1.0%, and 4.3% for the splanchnic group and 4.6%, 0.1%, 3.8%, and 4.7% for the non-splanchnic group. There was no significant different distribution of (ScvO_2 _- SvO_2_) between survivors and non-survivors in either the splanchnic group (*P *= 0.23) or the non-splanchnic group (*P *= 0.13) (Figure 3).

### Oxygen extraction ratio

**Figure 4:**
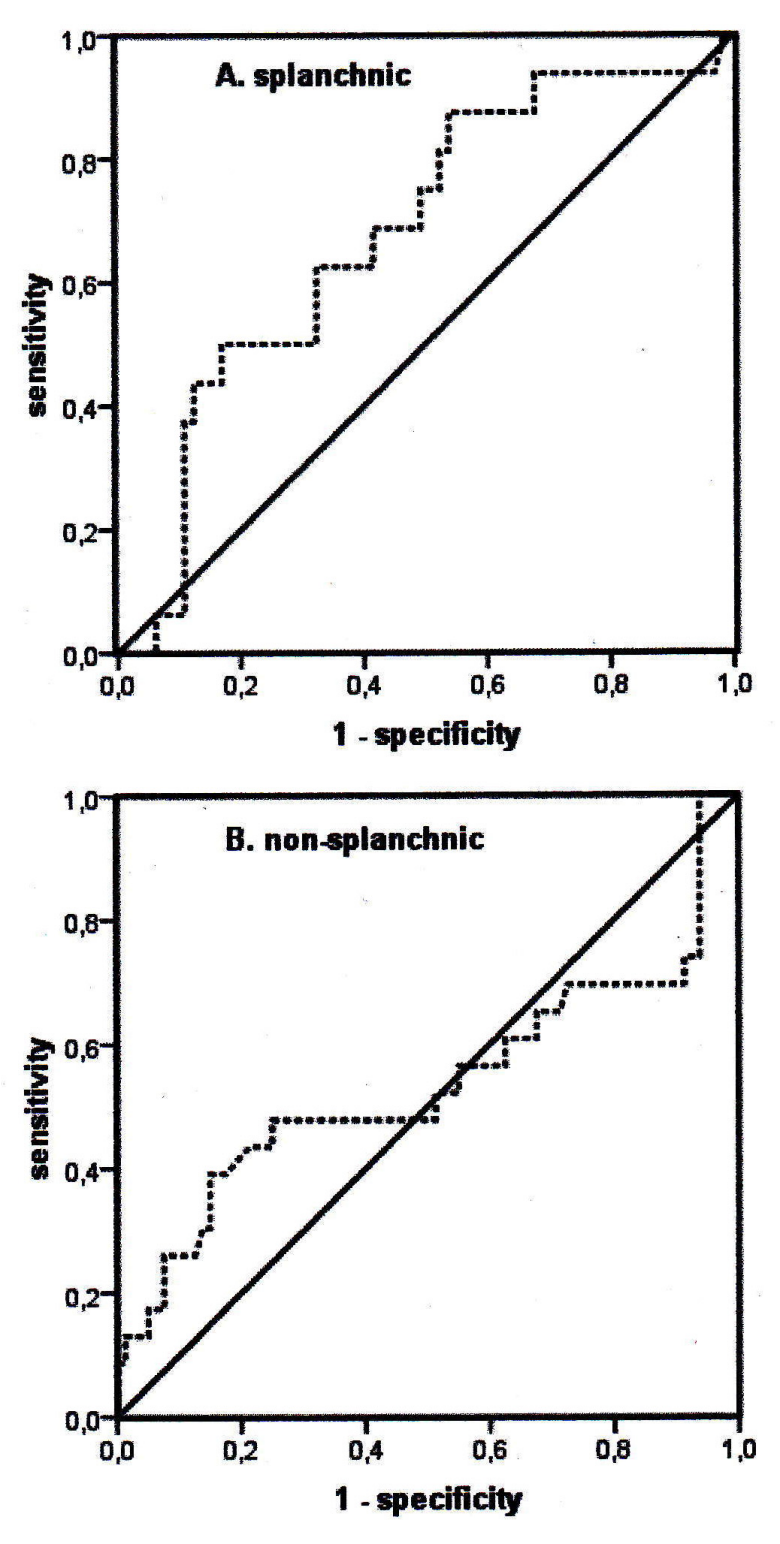
**Receiver operating characteristic curves of oxygen extraction ratio for the splanchnic and non-splanchnic groups**. The area under the curve (AUC) in the splanchnic group was not significantly larger than AUC in the non-splanchnic group (0.67 versus 0.55; *P *= 0.20).

The O_2_ER in the splanchnic group was similar to the O_2_ER in the non-splanchnic group (0.23 ± 0.07 versus 0.24 ± 0.09; *P *= 0.23). Figure 4 shows the ROC curves of O_2_ER for the splanchnic and non-splanchnic groups. Optimal values of O_2_ER were 0.22 (sensitivity = 0.46, specificity = 0.87) for the non-splanchnic group and 0.31 (sensitivity = 0.85, specificity = 0.40) for the splanchnic group. These curves represent the reliability of the O_2_ER as a predictor of in-hospital mortality. The area under the curve (AUC) in the splanchnic group was not significantly larger than the AUC in the non-splanchnic group (0.67 versus 0.55; *P *= 0.20).

## Discussion

We could confirm our hypothesis that ScvO_2 _does not reliably predict SvO_2 _in patients with severe sepsis: the agreement of ScvO_2 _and SvO_2 _was clinically not adequate. The difference between ScvO_2 _and SvO_2 _varied according to the level of ScvO_2 _and was the greatest in low (< 60%) and high (> 80%) ranges. In patients with severe sepsis or septic shock, the difference between ScvO_2 _and SvO_2 _appears not to be a fixed one and does not seem to be predictive for in-hospital mortality. Finally, the difference between ScvO_2 _and SvO_2 _is independent of several hemodynamic variables, with the exception of O_2_ER.

The bias was small, and ScvO_2 _was consistently larger than SvO_2_. However, this consistent bias also implies a greater relative error for SvO_2 _values at lower ScvO_2 _values. Additionally, the wide limits of agreement between ScvO_2 _and SvO_2 _are unacceptably wide and independent of time point. The widely assumed 5% difference between ScvO_2 _and SvO_2 _[1,8,9] seems not to be consistent in patients with severe sepsis or septic shock. A variety of factors influence the difference between both variables in patients with sepsis: mixing of the less saturated blood from the coronary sinus in the right atrium, sepsis-related vasodilatation, heterogeneity of flow within and between organs, and decreased cerebral oxygen uptake during sedation. On the basis of the present study, the net effect of these factors seems unpredictable. Our results seem concordant with earlier findings [6,8,16]. The first study described a small heterogeneous group of patients with septic shock. ScvO_2 _was consistently higher than SvO_2_, and the limits of agreement were equally wide. Moreover, the difference between ScvO_2 _and SvO_2 _varied according to the level of ScvO_2 _and deviated in the extreme ranges (60% < ScvO_2 _> 80%) [6]. The lower range (venous saturations of less than 60%) is clinically of the greatest interest because the patients admitted with such low venous saturations are the ones who could possibly benefit from ScvO_2_-guided therapy [4]. With the results of the present study in mind, the clinician should be aware of the large variability between ScvO_2 _and SvO_2_. Clinically important, this large variability was already present on admission (T = 0). At this time point, the first decisions on how to resuscitate and on what goals should be achieved are made. Such large uncertainty in estimating SvO_2 _by ScvO_2 _is unlikely to be suitable for protocol-guided resuscitation in which decreases in SvO_2 _or ScvO_2 _may trigger therapeutic interventions. Normalization of ScvO_2 _after resuscitation will not automatically imply normalization of SvO_2_.

If the individual values of ScvO_2 _and SvO_2 _do not agree, could this be different for the trends of ScvO_2 _and SvO_2_? In anesthetized subjects who underwent elective neurosurgery, measurement of oxygen saturations was performed in various hemodynamic conditions. It was concluded that for clinical purposes the trend of ScvO_2 _may be substituted for the trend of SvO_2 _[17]. In the present study, however, we found wide limits of agreement between the change of ScvO_2 _and the change of SvO_2 _in critically patients. As for the absolute values of ScvO_2 _and SvO_2_, substitution of the change of ScvO_2 _for the change of SvO_2 _in patients with sepsis is therefore undesirable. This is in concordance with earlier findings in patients with cardiogenic or septic shock: changes in ScvO_2 _and SvO_2 _did not follow the line of perfect agreement, and ScvO_2 _and SvO_2 _were not considered to be interchangeable [18].

Another issue is whether an ScvO_2 _of 70% as a treatment goal in sepsis or septic shock after resuscitation may be considered useful. In a study by Reinhart and colleagues [5], ScvO_2 _was measured continuously in critically ill patients for an average of 42 hours. More than 87% of the values in non-survivors and 95% of the values in survivors were above 70%. This difference was significant. Average time per patient below the cutoff value was twice as long in non-survivors [5]. In the present study, ScvO_2 _values in non-survivors fell more frequently below the cutoff value of 70% compared with survivors, and SvO_2 _values below 65% were more frequently found in non-survivors compared with survivors. Our data suggest that, after the first hours of resuscitation, monitoring of venous oxygen saturations could still be clinically relevant.

More recently, Gutierrez and colleagues [10] described an association between a positive (ScvO_2 _- SvO_2_) and ICU survival in critically ill patients. A significantly greater number of survivors had an (ScvO_2 _- SvO_2_) of at least 0 compared with non-survivors. The difference between ScvO_2 _and SvO_2 _became increasingly positive in survivors from initial to final measurement. The authors suggested that this may be associated with clinical recovery, perhaps reflecting a greater rate of O_2 _utilization [10]. A similar trend was observed in post-operative cardiac patients [19]. Although we noted that (ScvO_2 _- SvO_2_) was more frequently positive in survivors and that O_2_ER correlated with (ScvO_2 _- SvO_2_), we found no significant difference in distribution of (ScvO_2 _- SvO_2_) between survivors and non-survivors. Our results could not confirm a greater rate of O_2 _utilization in survivors as suggested by Gutierrez and colleagues [10]. However, it is possible that the number of measurements in our study was not sufficient to detect a difference in distribution of (ScvO_2 _- SvO_2_).

Secondary analysis showed that the inconsistent difference between ScvO_2 _and SvO_2 _is independent of sepsis origin. There was no significant difference between the mean (ScvO_2 _- SvO_2_) of the two groups, and the limits of agreement were wide both for the absolute values and for the changes in ScvO_2 _and SvO_2_. SvO_2 _values were higher in the splanchnic group compared with the non-splanchnic group for a certain ScvO_2 _value. This phenomenon could be explained by sepsis-related vasodilatation in the digestive tract. Despite heterogeneity of flow within and between various organs in patients with splanchnic sepsis [20], this leads to diminished oxygen consumption, which results in a higher SvO_2_. Apparently, a normal SvO_2 _does not rule out the presence of limited oxygen consumption in the splanchnic region [7]. Moreover, we found no difference in O_2_ER between the splanchnic and non-splanchnic groups. This suggests less oxygen utilization in the digestive tract than could be expected on the basis of the assumption that in all septic patients the difference between ScvO_2 _and SvO_2 _equals 5%.

This study has limitations. First, all patients were sedated and mechanically ventilated and none of them was in hemorrhagic shock. Our findings may not be generalized to patients who are less critically ill or those with hemorrhagic shock. Also, owing to intubation, ScvO_2 _values could have been relatively high in relation to disease severity [21]. Second, we investigated ICU patients, who may have been in a later stage of sepsis; timing of measurements was probably not all in the same stage of critical illness. Third, in this study, ScvO_2 _and SvO_2 _values did not change between different time points as a result of a protocolized intervention: conclusions on independence of time points are of limited value. However, measurements were conducted within individual patients: each subject served as his or her own control. Finally, we used the proximal port of the catheters as a surrogate of ScvO_2_. A more distal location in the right atrium allows mixing of superior and inferior caval vein blood, and some ScvO_2 _measurements might have been influenced by this. Nevertheless, our results are consistent with those of previous studies in which a similar technique was used [6,8,10].

## Conclusions

We conclude that ScvO_2 _does not reliably predict SvO_2 _in patients with sepsis, independently of sepsis origin. Assuming a consistent 5% difference between ScvO_2 _and SvO_2 _can lead to erroneous clinical decisions. The change of ScvO_2 _compared with the change of SvO_2 _is not more reliable than the exact numerical values in this context. Finally, a positive (ScvO_2 _- SvO_2_) value is not associated with improved outcome in patients with sepsis. The abovementioned conclusions apply to sepsis of either splanchnic or non-splanchnic origin.

## Key messages

• Central venous saturation (ScvO_2_) does not reliably predict mixed venous saturation (SvO_2_) in patients with sepsis, independently of sepsis origin.

• The change of ScvO_2 _compared with the change of SvO_2 _is not more reliable than the exact numerical values in patients with sepsis.

## Abbreviations

AUC: area under the curve; CCO: continuous cardiac output; ICU: intensive care unit; MCL: Medical Center Leeuwarden; MH: Martini Hospital; O_2_ER: oxygen extraction ratio; PAC: pulmonary artery catheter; ROC: receiver operating characteristic; ScvO_2_: central venous saturation; SvO_2_: mixed venous saturation.

## Competing interests

The authors declare that they have no competing interests.

## Authors' contributions

PAvB drafted the manuscript, participated in its design and coordination, and performed statistical analysis. JvI was responsible for acquisition of patient data in MH and helped to draft the manuscript. ECB and NDH participated in the design of the study and helped to draft the manuscript. HG advised in statistical analysis and helped to draft the manuscript. MK was responsible for acquisition of patient data in MCL. PES provided general support and helped to draft the manuscript. MAK conceived of the study and participated in its design and coordination and helped to draft the manuscript. All authors read and approved the final manuscript.

## References

[B1] Dellinger RP Carlet JM Masur H Gerlach H Calandra T Cohen J Gea-Banacloche J Keh D Marshall J Parker MM Ramsay G Zimmerman JL Vincent JL Levy MM for Surviving Sepsis Campaign Surviving Sepsis Campaign guidelines for management of severe sepsis and septic shock Crit Care Med 2004 32 858 873 10.1097/01.CCM.0000117317.18092.E4 15090974

[B2] Edwards JD Oxygen transport in cardiogenic and septic shock Crit Care Med 1991 19 658 663 10.1097/00003246-199105000-00012 2026028

[B3] Krafft P Steltzer H Hiesmayr M Klimscha W Hammerle AF Mixed venous oxygen saturation in critically ill septic shock patients. The role of defined events Chest 1993 103 900 906 10.1378/chest.103.3.900 8449089

[B4] Rivers E Nguyen B Havstad S Ressler J Muzzin A Knoblich B Tomlanovich M for the Early Goal-Directed Therapy Collaborative Group Early goal-directed therapy in the treatment of severe sepsis and septic shock N Engl J Med 2001 345 1368 1377 10.1056/NEJMoa010307 11794169

[B5] Reinhart K Kuhn HJ Hartog C Bredle DL Continuous central venous and pulmonary artery oxygen saturation monitoring in the critically ill Intensive Care Med 2004 30 1572 1578 10.1007/s00134-004-2337-y 15197435

[B6] Varpula M Karlsson S Ruokonen E Pettilä V Mixed venous oxygen saturation cannot be estimated by central venous oxygen saturation in septic shock Intensive Care Med 2006 32 1336 1343 10.1007/s00134-006-0270-y 16826387

[B7] Dahn MS Lange MP Jacobs LA Central mixed and splanchnic venous oxygen saturation monitoring Intensive Care Med 1988 14 373 378 10.1007/BF00262891 3403769

[B8] Chwala LS Zia H Guttierez G Katz NM Seneff MG Shah M Lack of equivalence between central and mixed venous oxygen saturation Chest 2004 126 1891 1896 10.1378/chest.126.6.1891 15596689

[B9] Rivers E Mixed vs central venous oxygen saturation may be not numerically equal, but both are still clinically useful Chest 2006 129 507 508 10.1378/chest.129.3.507 16537845

[B10] Gutierrez G Comignanni P Huespe L Hurtado FJ Dubin A Jha V Arzani Y Lazzeri S Sosa L Riva J Kohn W Suarez D Lacuesta G Olmos D Mizdraji C Ojeda A Central venous to mixed venous blood oxygen and lactate gradients are associated with outcome in critically ill patients Intensive Care Med 2008 34 1662 1668 10.1007/s00134-008-1128-2 18542920

[B11] Levy MM Fink MP Marshall JC Abraham E Angus D Cook D Cohen J Opal SM Vincent JL Ramsay G 2001 SCCM/ESICM/ACCP/ATS/SIS International Sepsis Definitions Conference Intensive Care Med 2003 29 530 538 12664219 10.1007/s00134-003-1662-x

[B12] Knaus WA Draper EA Wagner DP Zimmerman JE APACHE II: a severity of disease classification system Crit Care Med 1985 13 818 829 10.1097/00003246-198510000-00009 3928249

[B13] Friedman LM Fuberg CD DeMets DL Fundamentals of Clinical Trials 1998 3 New York, NY; Springer-Verlag 111

[B14] van Beest PA Hofstra JJ Schultz MJ Boerma EC Spronk PE Kuiper MA The incidence of low venous oxygen saturation on admission in the ICU: a multicenter observational study in the Netherlands Crit Care 2008 12 R33 10.1186/cc6811 18318895 PMC2447553

[B15] Bland JM Altman DG Agreement between methods of measurement with multiple observations per individual J Biopharm Stat 2007 17 571 582 10.1080/10543400701329422 17613642

[B16] Martin C Auffray JP Badetti C Perin G Papazian L Gouin F Monitoring of central venous oxygen saturation versus mixed venous oxygen saturation in critically ill patients Intensive Care Med 1992 18 101 104 10.1007/BF01705041 1613187

[B17] Dueck MH Klimek M Appenrodt S Weigand C Boerner U Trends but not individual values of central venous oxygen saturation agree with mixed venous oxygen saturation during varying hemodynamic conditions Anesthesiology 2005 103 249 257 10.1097/00000542-200508000-00007 16052106

[B18] Ho KM Harding R Chamberlain J Bulsara M A comparison of central and mixed venous oxygen saturation in circulatory failure J Cardiothorac Vasc Anesth 2010 24 434 439 10.1053/j.jvca.2007.10.011 18834813

[B19] Sander M Spies CD Foer A Weymann L Braun J Volk T Grubitzsch H von Heymann C Agreement of central venous saturation and mixed venous saturation in cardiac surgery patients Intensive Care Med 2007 33 1719 1725 10.1007/s00134-007-0684-1 17525841

[B20] Boerma EC van der Voort PHJ Spronk PE Ince C Relationship between sublingual and intestinal microcirculatory perfusion in patients with abdominal sepsis Crit Care Med 2007 35 1055 1060 10.1097/01.CCM.0000259527.89927.F9 17334238

[B21] Hernandez G Peña H Cornejo R Rovegno M Retamal J Navarro JL Aranguiz I Castro R Bruhn A Impact of emergency intubation on central venous oxygen saturation in critically patients: a multicenter observational study Crit Care 2009 13 R63 10.1186/cc7802 19413905 PMC2717418

